# Measuring research impact in medical research institutes: a qualitative study of the attitudes and opinions of Australian medical research institutes towards research impact assessment frameworks

**DOI:** 10.1186/s12961-018-0300-6

**Published:** 2018-03-16

**Authors:** Simon Deeming, Penny Reeves, Shanthi Ramanathan, John Attia, Michael Nilsson, Andrew Searles

**Affiliations:** 1 Hunter Medical Research Institute, Lot 1, Kookaburra Circuit, New Lambton Heights, NSW 2305 Australia; 2 School of Medicine and Public Health, The University of Newcastle, University Drive, Callaghan, NSW 2308 Australia; 3 Department of Medicine, John Hunter Hospital, Hunter New England Local Health District, New Lambton Heights, NSW 2305 Australia

**Keywords:** Qualitative research, Medical research institutes, Australia, Research assessment, Research impact

## Abstract

**Background:**

The question of how to measure, assess and optimise the returns from investment in health and medical research (HMR) is a highly policy-relevant issue. Research Impact Assessment Frameworks (RIAFs) provide a conceptual measurement framework to assess the impact from HMR. The aims of this study were (1) to elicit the views of Medical Research Institutes (MRIs) regarding objectives, definitions, methods, barriers, potential scope and attitudes towards RIAFs, and (2) to investigate whether an assessment framework should represent a retrospective reflection of research impact or a prospective approach integrated into the research process. The wider objective was to inform the development of a draft RIAF for Australia’s MRIs.

**Methods:**

Purposive sampling to derive a heterogeneous sample of Australian MRIs was used alongside semi-structured interviews with senior executives responsible for research translation or senior researchers affected by research impact initiatives. Thematic analysis of the interview transcriptions using the framework approach was then performed.

**Results:**

Interviews were conducted with senior representatives from 15 MRIs. Participants understood the need for greater research translation/impact, but varied in their comprehension and implementation of RIAFs. Common concerns included the time lag to the generation of societal impacts from basic or discovery science, and whether impact reflected a narrow commercialisation agenda. Broad support emerged for the use of metrics, case study and economic methods. Support was also provided for the rationale of both standardised and customised metrics. Engendering cultural change in the approach to research translation was acknowledged as both a barrier to greater impact and a critical objective for the assessment process. Participants perceived that the existing research environment incentivised the generation of academic publications and track records, and often conflicted with the generation of wider impacts. The potential to improve the speed of translation through prospective implementation of impact assessment was supported, albeit that the mechanism required development.

**Conclusion:**

The study found that the issues raised regarding research impact assessment are less about methods and metrics, and more about the research activities that the measurement of research translation and impact may or may not incentivise. Consequently, if impact assessment is to contribute to optimisation of the health gains from the public, corporate and philanthropic investment entrusted to the institutes, then further inquiry into how the assessment process may re-align research behaviour must be prioritised.

## Background

The reasons driving the international push for assessment of the impacts from health and medical research (HMR) are significant, sustained and generic across many Organisation for Economic Co-operation and Development nations [1–5]. First, given the extensive reliance of HMR upon tax exemptions and government investment, the level of evidence required to substantiate on-going support against competing budget priorities has risen. Second, rising healthcare demands amid constrained health budgets provides an imperative for research to generate improvements in health outcomes with the same or less public expenditure [6]. Third, Governments increasingly expect benefits for the wider economy to be realised via the commercialisation of research into medical services, pharmaceuticals and medical devices [7]. Finally, to realise all these goals it is necessary for HMR to challenge the unproductive research practices that hamper translation and health impact [8]. As a consequence, the question of ‘how’ to assess the impact from investment in HMR remains a significant issue.

Research Impact Assessment Frameworks (RIAFs) provide a conceptual framework and methods against which the translation and impact of HMR can be assessed. As such, these frameworks potentially offer a mechanism to contribute to the realisation of the above goals [5]. This study represents one component of a larger research project to develop a draft RIAF for Australia’s medical research institutes (MRIs) [5]. Australia supports approximately 70 independent MRIs, which collectively facilitate the work of approximately 10,100 health and medical researchers [9]. The teaching responsibilities of universities and/or the challenges facing research fields for which translation outcomes may carry less relevance complicates impact assessment within educational institutions and for disciplines outside of HMR. The focus upon MRIs clarifies the analysis by reducing the issue to the explicit translation and impact considerations from research activity in a discipline, HMR, which ultimately seeks to improve human health.

Prior components of this study included a literature review of the objectives provided for different RIAFs, identification of 25 RIAFs that have been used to assess the impact of HMR, and a review of the capacity of these frameworks to realise the specified objectives [5]. This paper outlines the qualitative component of the research project. The aims include (1) to elicit the views of MRIs regarding objectives, definitions, methods, barriers, potential scope and attitudes towards RIAFs, and (2) to investigate whether an assessment framework should represent a retrospective reflection of research impact or a prospective approach integrated into the research process. The broader objective was to inform on the guiding principles for development of a draft RIAF for Australia’s MRIs. Subsequent research will draw the implications from the results of this qualitative research and the prior study to inform upon guiding principles for a RIAF tailored to Australia’s MRI sector.

### Definitions

Given that the participants in this research used the terms interchangeably, for the purpose of this paper, the term metrics and indicators are interchangeable.

## Methods

This study comprised primary analysis of qualitative data collected via semi-structured interviews with representatives from 15 MRIs and five key stakeholders. Ethical approval was obtained from the University of Newcastle Human Research Ethics Committee, approval number H-2015-0250.

### Samples and procedures

The target population comprised all Australian MRIs. Depending upon the categorical definition, Australia supports approximately 70 independent MRIs. Membership of the Association of Australian Medical Research Institutes was selected as the sampling frame/inclusion criteria (*n* = 46), as it excluded organisations for whom research did not represent their core function, e.g. ANZ College of Anaesthetists. Participating organisations (*n* = 15) were identified using purposeful sampling to provide a heterogeneous sample of MRIs, based on size, research specialisation, their relationship to researchers, i.e. direct employment or facilitation, their progress in implementing research assessment frameworks, their affiliation with hospitals and/or universities, and their geographical distribution across the six Australian states [10, 11].

From the original 15 institutes approached to participate, one could not be contacted and one declined to participate; both were replaced with an equivalent organisation. The 15 participating MRIs are listed in Appendix 1 and their profiles outlined in Table 1. The sample represented 33% of the target population within the sampling frame.

**Table 1:** MRI participant profile (organisations) (*n* = 15)

	Participants (N, %^c^)
Size^a^	
Large (400+ researchers)	7 (47%)
Medium (100–399 researchers)	6 (40%)
Small (0–99 researchers)	2 (13%)
Research specialisation (spectrum, not disease or population)^b^	
Narrow	5 (33%)
Medium	6 (40%)
Broad	4 (27%)
Relationship to researchers^a^	
Majority employed directly	12 (80%)
Majority employed by affiliated organisations (universities, hospitals, etc.)	3 (20%)
Progress implementing Research Impact Assessment^b^	
None	4 (27%)
Initiated	8 (53%)
Advanced	3 (20%)
Affiliation to Universities or Health Districts^b^	
Affiliated mainly with University	8 (53%)
Affiliated mainly with Health District	0 (0%)
Affiliated with both	6 (40%)
No affiliations	1 (7%)
State^a^	
New South Wales	4 (27%)
Queensland	2 (13%)
South Australia	1 (7%)
Tasmania	1 (7%)
Victoria	6 (40%)
West Australia	1 (7%)

^a^Categorisation known prior to participant selection

^b^Categorisation confirmed at participant interviews

^c^May exceed 100% due to rounding

Participating institutes and identified participants were provided with an Organisation and Participation Information Statement and Organisation and Participant Consent Forms. Written consent from both organisations and participants were obtained prior to each interview. The institutes were requested to identify relevant participants based on the following criteria:

- Administrators or researchers charged explicitly with the development of frameworks, or responsible more generally for strategy, stakeholder management, reporting and related research management
- Researchers who would be informed by the framework, but are not directly involved in the related strategy or framework development.

The institutes identified either one or two participants for each interview. The roles of the 18 participants are summarised in Table 2.

**Table 2:** Participant profile (individuals representing Medical Research Institutes; 1 or 2 participants per institute) (*n* = 18)

Respondent roles	Participants (N, %)
Chief Executive Officer	3 (17%)
Chief Operating Officer	5 (28%)
Commercial Manager	2 (11%)
Research Manager/Knowledge Translation Manager	4 (22%)
Chief Scientist/Senior/Mid-career Researcher	4 (22%)

The five participating stakeholders included the main research funding bodies, the Australian Research Council and the Australian National Health and Medical Research Council (NHMRC), due to their critical role in Australia’s HMR. Other stakeholders were identified via network sampling, where new participants were recommended by existing participants [12]. They included a representative from the Association of Australian Medical Research Institutes, an expert in research commercialisation and an international expert in research assessment methods. The participating stakeholders are listed in Appendix 1.

### Data collection

Semi-structured interviews were conducted on a 1:1, 2:1 and 2:2 format (with the first number representing the number of interviewers and the second representing the number of interviewees) with representatives from the MRIs. All interviews were conducted by hands-free teleconference, which provided for the collection of supporting field notes. Audio recordings were made and transcribed verbatim for 13 of the 15 MRI interviews. Two MRIs elected not to be recorded, but consented to field notes, which were immediately transcribed. Field notes were taken for all MRI interviews to consolidate the meaning, emphasis and context. Participants were encouraged to expand on areas of interest beyond the interview structure. The interviews ranged in length from 55 to 110 min. Two of the longer interviews were conducted over two sessions.

Semi-structured interviews were also conducted on a 1:1, 2:5 and 2:3 format with five key stakeholders. At their request, face-to-face interviews were conducted with the Australian Research Council, the NHMRC and the commercialisation expert. Other stakeholder interviews were conducted by teleconference. Three of the five stakeholders elected not to be recorded to provide for a frank conversation. Due to the limited interview time available, the NHMRC took questions on notice, collated responses from the participants and provided responses in text.

All interviews were conducted in the period from December 2015 to May 2016.

### Survey instrument

The interview guide used to structure the discussion is provided in Appendix 2. Prior to this study, the authors had conducted research to develop a Framework To Assess the Impact from Translational health research, or FAIT, at the Hunter Medical Research Institute [13]. The interview guide was informed by this a priori knowledge regarding the key issues, while also providing the opportunity for new areas of investigation.

### Determining sample size

The sample size was determined by the project scope and research budget, rather than theme saturation. As such, the study sought to identify the majority of pertinent issues rather than all issues.

### Data analysis

A thematic analysis was conducted of the qualitative data collected during the interviews. To provide a clear audit trail to participants’ verbatim comments, the thematic analysis followed the framework method [14]. Following transcription of the interviews and the field notes, the transcripts were reviewed to familiarise the researchers with potential themes. Given that the interviews were conducted by researchers with substantial experience in this field and that the form and content of the survey instrument was heavily informed by prior interviews with medical and health researchers from the Hunter Medical Research Institute [13], the analysis commenced with a theme structure founded on the prior research. The data was indexed using the thematic framework to code the data. Coding followed a combination of deductive and inductive approaches. Deductive coding was used to index insights into known themes. However, the semi-structured open-ended interviews also provided for new areas of investigation to arise. In this instance, a more inductive approach was adopted to identify initial categories, refine categories and allocate the un-coded information. The analyses identified emergent themes through repeated review of coding to assess reliability of previously coded text and identify new themes. The data was subsequently grouped to themes and sub-themes according to the codes. The final stage comprised explanation of the findings with respect to the aims of the study.

### Reflexivity

During the period over which the interviews were conducted, as a result of parallel research and the outcomes from the earlier interviews, the weight of the researchers’ focus developed from questions of definition, opinion and method towards questions of why, objectives and the capacity to orientate/facilitate research practice. The result of this reflection did not alter the research aims, nor the interview questions, but served to shift the weight of time invested probing the respective issues. For example, the interview time investigating consistent responses regarding the definition for ‘research impact’ was minimised, while discussion regarding the potential for research impact metrics to effect change, address perverse incentives or optimise of the value from research investment was encouraged and expanded, where appropriate.

### Maintaining research quality

The Consolidated Criteria for Reporting Qualitative Research (COREQ) framework was used to define the content of this paper [15]. Transparency was provided to the study via detailing of the sample procedures and to the study content via the use of direct quotes. Strategies used to address credibility included recording interviews; use of complimentary field notes; provision for non-recording to aid openness; prompt transcription of both recorded interviews and field notes; frequent discussion of findings between SD, AS and PR; enabling participants to elaborate along their own line of interest; searching the data for conflicting patterns; and a clear audit trail between transcription and analysis. Confirmability was addressed by rigorous review of interview transcripts, the codes used to identify themes and the draft findings, and triangulation of the results with the results from the prior literature review and capability analysis [5]. In addition, transferability was addressed via detailed presentation of the population, sample methods, instruments and analytical methods.

## Results

**Fig. 1:**
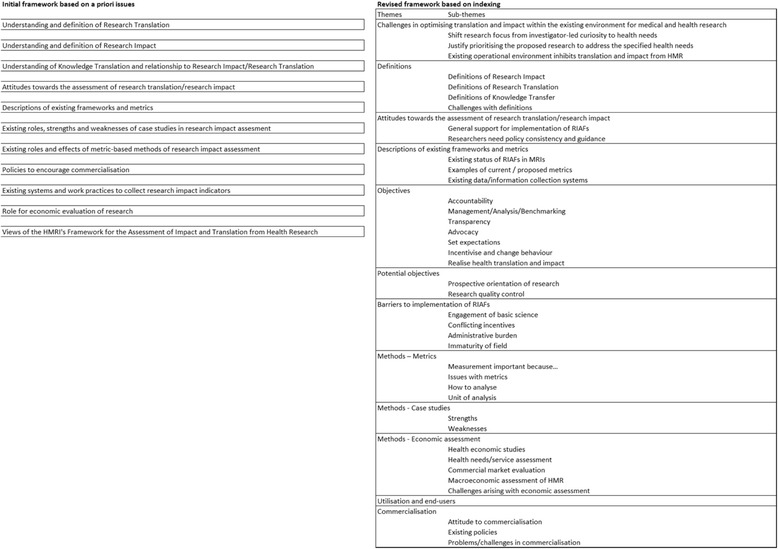
Stages of framework analysis for Medical Research Institute representatives’ and stakeholders’ views regarding research impact assessment

The framework analysis commenced with 11 issues and evolved to 12 themes and 40 sub-themes (Fig. 1).

### Challenges for translation and impact within existing HMR practice

#### Shift research focus from investigator-led curiosity to health needs

From the perspective of increasing research translation and generating greater research impact, the participants raised a number of challenges with the existing approach to HMR. Firstly, the weight of research focus was perceived to be misaligned with the community’s health requirements. Relative to other international health funders, the approach to Australia’s HMR funding was seen as driven by investigator-led curiosity rather than health needs.

“[Consider] *the Canadian Institutes of Health - fair amount of their budget for health and medical research which is explicitly driven by priority, by disease and population priority, whereas ours isn't at all.*” Chief Operating Officer (COO), MRI, 2016

While these challenges remain, some respondents emphasised that the context was changing with health needs increasingly shaping the research agenda.

“*Aboriginal Health Services - we've negotiated with primary care as well as tertiary care and with the communities and with the Aboriginal-led community or organisations that run primary care, what they want. In that environment we can only do what's specified by the community, so they know exactly what they want out of the research.*” COO, MRI, 2016

#### Justify prioritising the proposed research to address the specified health needs

The shift to a health needs agenda introduced an additional concern, particularly amongst funders, that substantial health needs were used to justify a research requirement, without informing on the nature of the research.

“*Type II diabetes* [Diabetes mellitus type 2] *…we don't really need to know more about the disease pattern physiology. We understand it. Eat less. Exercise more. But that doesn't work so we need more research about how to do that and whether that means it's a tax on soft drinks or whatever. ... I think some of the projections for the diabetes problem is that by 2050 it would consume the whole federal budget, not just the health budget.*” Chief Executive Officer (CEO), MRI, 2016

#### Existing operational environment inhibits translation and impact from HMR

It was a commonly held view that the academic system, including research grants and academic promotions, provides incentives skewed to publications, bibliometric impact factors and further grant success, rather than research translation and the generation of wider ‘impact’.

“*What drives any research; it’s survival. In the environment we are in, survival. It’s such a competitive environment. This is what is on top of their mind. Rightly or wrongly.*” Researcher, MRI, 2016

“*People will do whatever the funding system rewards them for doing…it's about my individual success as a publisher…that's what I'll get rewarded for within the grant system so…I'll become a world expert on this discrete molecule that no-one cares about.*” CEO, MRI, 2016

“*What worries me most is the 85 percent or whatever the number is of papers that are never cited.*” CEO, MRI, 2016

It was considered that research grant applications continue to prioritise research quality and academic track records. While changing, relatively limited weight was given to research translation plans or track records of translation and impact. If grant review panels do not value, or have the capacity to value, research translation, then the incentives are misaligned with improved translation. This did not imply that the participants placed no value on scientific rigour, research quality or traditional academic capabilities, but rather noted the lack of value given to translation. For example:

“*It’s almost impossible to get academic peer review panels to value anything that anybody has done in the private sector that doesn’t involve publication.*” CEO, MRI, 2016

“*Take NHMRC grant review panels – expensive oncology drug $50,000 pa, trials by pharmaceutical companies eight monthly cycles; researchers believe didn’t need that many; …simple trial randomised people into six or eight cycles of the drug; cost savings PBS greater than the cost for the trials pays for the whole study; we didn’t fund it because… bit boring. Everyone agreed* [it was a] *sensible thing to do, but it was a bit boring.*” CEO, MRI, 2016

Existing initiatives to incorporate translation into the funding process were also considered ineffective by some.

“*NHMRC Development Grants - Not very effective mechanism to promote commercialisation thinking, just knowing some applications. Things are changing, …*[but] *it's just been research to see if this is another way of funding what they've always done; made little changes to try and make them look like they're doing what they are supposed to be doing to tick the box.*” Commercialisation Manager, MRI, 2016

### Definitions

None of the MRI participants could clearly reference an institutional definition of research translation, research impact or knowledge transfer. While most volunteered a personal definition for research translation and research impact, only one participant held a preconceived delineation between the terms.

#### Research impact

Albeit acknowledged as a legacy interpretation, research impact was presented by some as the traditional academic measures of publications, citations, other bibliometrics, grants and awards. The more contemporary interpretation of research impact reflected the challenge “*to change lives*”, extend lifespans and improve quality of life. Numerous participants referred to the constitution or strategic plan of their institute as embodying the research impact definition – “*research impact is health impact*” (CEO, MRI). Many noted that health impact at the population level “*set a very high bar*” (Knowledge Transfer (KT) Manager, MRI) and favoured interim definitions, including improved therapy or diagnostics, commercialisation, influence upon policy, new knowledge or the take-up of research findings by end-users.

#### Research translation

In the broader sense, some participants interpreted research translation as the cure, prevention and treatment of disease, as distinct from publications. As such, there was no clear distinction from other respondent’s definition of research impact. However, research translation was more commonly seen as a prelude to health impact. Examples presented include the translation of research into clinical practice, sometimes via clinical trials, into policy, into education and training, into health service changes, as commercialisation, or via the development of potential new treatments or diagnostics.

#### Knowledge transfer (KT)

Of the few participants that presented a definition, KT was disparately described as the movement of new knowledge into policy, the legal transfer of technology/molecule intellectual property or the improvement of capability via education and training. KT was also presented conceptually, as part of the mechanism of the translation process, but also more specifically, as the involvement of a third party to progress translation via commercialisation or alternative pathways.

#### Challenges with definitions

The results clearly demonstrate that there is a lack of clarity and substantial overlap between the different interpretations of the three terms.

“*…a plethora of different terminologies and I think by and large, people are increasingly talking about the same things even though they’re still using quite different words.*” KT Manager, MRI, 2016

“*The definitions are largely a semantic issue, the question is really ‘How we do the best research* [to generate translation]*?’*” KT Manager/Clinician Researcher, MRI, 2015

Irrespective of the definition, the participants raised a number of challenges that the scope of the definitions need to address, including the need to include drug discovery/basic science; acknowledgement that the translation pathway is not linear, but messy; and definitions that address the flawed interpretation of research translation as simply the dissemination of research findings.

“*…the difficulty with the term research translation is, it sort of has a bit of an implied, we do the research and then we disseminate the findings*”, “*…you heard at the conference translation was looked at as, ‘This is what we do after we do the research.’*” KT Manager/Clinician Research, MRI, 2015

### Attitudes towards the assessment of research translation/research impact

#### General support for implementation of RIAFs

Of the MRI participants interviewed, all provided general support for the implementation of RIAFs, either explicitly or implicitly via their development or implementation of a RIAF. It was argued that taxpayer funds provided an obligation to demonstrate the contribution of research

“*‘We’d better do translation because NHMRC expects us to.’ I think it's moving much more into that loftier sense that actually we want to do research that has a really big impact.*” KT Manager/Clinician Researcher, MRI, 2015

“*On the whole I think our researchers are very receptive to the idea.*” Researcher, MRI, 2016

Attitudes towards assessment of research translation and impact were conflated with attitudes towards the measurement of research translation/impact. Assessment may or may not include measurement. Case studies, for example, may be used for assessment, but do not represent measurement. Attitudes to measurement are addressed further under the Methods – Metrics theme.

#### Researchers need policy consistency and guidance

The potential for a new government to change the direction of research policy was also noted. Sustainability was sought across partisan lines and political cycles before researchers would fully commit. Participants reported that many researchers did not have the knowledge or guidance of how to think about or demonstrate research impact. The experience of some participants, as reviewers of partnership grant applications, was that the content ranged “*from absolute waffle to really precise accounts*” (CEO, MRI, 2016). A clear sub-theme emerged that researchers were generally keen to make an impact on health, but needed significant guidance. A template framework, guidelines for researchers and formalised networks were requested to progress the development and implementation of RIAFs across the MRI community.

### Descriptions of existing frameworks and metrics

#### Existing status of RIAFs in MRIs

Participant descriptions of their institute’s progress implementing RIAFs could be categorised as none (*n* = 4), initiated (*n* = 8) and advanced (*n* = 3). The institutes reporting ‘advanced’ progress had undertaken, or were undertaking, a programme of investigation to determine the scope and content of a framework and had progressed to initial stages of implementation. The MRIs categorised as ‘initiated’ provided evidence of the collection, aggregation and reporting of outcomes beyond traditional academic metrics. Research translation and impact was commonly captured using metrics, albeit sometimes supplemented by case studies. The majority of non-academic metrics were captured as Key Performance Indicators (KPIs) for strategic plans, for performance evaluation and for reporting to Boards and state government medical infrastructure offices. Those classified as ‘none’ largely concentrated upon the collection of academic metrics for Excellence in Research Australia and the Higher Education Research Data Collection obligations.

#### Examples of current/proposed metrics

Participants were not asked to list current or proposed impact measures, but numerous metrics were provided as examples of academic impact, commercial translation, policy impact, engagement and community/social/economic impact. Academic impact metrics included traditional measures, such as bibliometric impact factors, Category 1 to 4 grants, international prizes and PhD completions, but also measures focussed upon the significance of research, such as citations within field, number of papers in top 1% of field, longevity of citations and editorial interest. The relative esteem of the journal was considered as a traditional metric, but also as a leading indicator of impact within the research community. Commercialisation metrics included patents, disclosures, milestone payments, licenses to commercialise, the measures included in the Australian National Survey of Research Commercialisation and “*everything through the commercialisation pipeline*” (Research Manager, MRI, 2016). Policy utilisation was measured by baseline and follow-up interviews and policy citations. While noted as important, only a few health-related metrics were quoted, including the number of clinical trials, citations in international or national clinical or governance guidelines, presentations to international clinical meetings, the number of clinician researchers and the number of clinician PhD students. Engagement metrics included the number of interactions with philanthropists and non-governmental organisations, positions on research committees and the existence of government engagement strategies. The few community/social/economic impacts raised included employment, economic activity and high school seminars.

#### Existing data/information collection systems

Aside from utilising common systems for the dissemination of traditional academic metrics, such as bibliometrics, the majority of data collection systems relied upon a combination of manual review and spreadsheets. The few MRIs that had made advanced progress towards the implementation of frameworks had made steps either in terms of building collection responsibilities into position descriptions or the development of potential tenders for data collection platforms.

### Objectives

A number of objectives were presented for the implementation of RIAFs, including accountability, transparency, management/analysis/benchmarking, advocacy, set expectations, incentivise and change behaviour, and to, more broadly, realise health translation and impact.

#### Accountability

The need to be accountable for research funding was presented both as a justification to external stakeholders and a challenge to established academic practice.

“*…very tight* [Federal Government] *budget; very difficult for them* [researchers]*. It’s not direct service provision…like healthcare or education; to justify to the community, they need evidence.*” Stakeholder, 2016

“[There is a] *sense of entitlement in academia…need to be more accountable.*” COO, MRI, 2016

#### Management/Analysis/Benchmarking

Participants expected RIAFs to inform management decisions regarding recruitment, funding allocation and facilities/equipment allocation. They wished to identify research areas that consistently fail to impact beyond academic outcomes. RIAFs were also seen as the means to analyse whether strategic initiatives to improve impact were proving effective, such as efforts to improve collaboration with clinicians, or the provision of biostatistics expertise. RIAFs were also anticipated to provide the capacity to benchmark impact, both internationally and between MRIs, with the potential to inform subsequent management decisions.

#### Transparency

The potential for a framework to provide transparency to both opportunities and problems was aligned with accountability and analysis.

#### Advocacy

The demonstration of translation and impact for advocacy to government, philanthropists or the wider community comprised another common objective for research impact assessment.

#### Set expectations

Impact frameworks were seen as important to set expectations and create a culture where entrepreneurship and the ability to influence policy were valued as well as academic measures.

#### Incentivise and change behaviour

The capacity for RIAFs to incentivise or change research behaviour was noted as an intended or experienced objective for RIAFs by six participants.

“[Our] *framework has effectively changed behaviour*” CEO, MRI, 2016

For those focussed upon research translation and impact, a level of frustration existed with the failure of their fellow researchers to pursue translation, while acknowledging that this resulted from incentives in the existing research system.

“*Presently no impetus for many in medical and health research to change*” KT Manager/Clinician Researcher, MRI, 2015

The ability that a RIAF provided to direct researchers beyond external drivers was presented as another reason. Without this ability to “*steer the ship*” (COO/General Manager Research Support, MRI, 2016), it was perceived that the MRIs were beholden to other incentives upon researchers that may not align with their translation objectives.

“*It’s a matter of re-internalising the process of deciding what's good, not do whatever the NHMRC funds and won't do whatever it doesn't fund*” COO, MRI, 2016

Frameworks were seen as a method to align researchers with translation by questioning their potential long-term impact and defining how their research aimed to contribute to the realisation of this goal.

“[Need to] *ensure that researchers think beyond their immediate research context*” Researcher, MRI, 2015

“*If your work is successful, ‘who's going to get better or not get sick?’ is a hard question for a cell biologist...* [but] *there has to be a clinical or therapeutic end point which is informing the work that they're doing.*” COO, MRI, 2016

#### Realise health translation and impact

Some participants stated the objective as the holistic goal to realise health translation and impact. While not detailed, this objective emphasises that the purpose for RIAFs can move beyond passive demonstration for accountability or advocacy purposes to more proactive intentions to generate health translation and impact.

### Potential objectives

#### Prospective orientation of research

This objective utilises a RIAF as a mechanism for researchers to prospectively scrutinise the potential impact of research. For example, an MRI’s metrics for research could capture engagement with potential ‘users’ of the anticipated research outputs at the inception stage of a project. None of the participants explicitly raised or had considered this potential objective for a RIAF. When prompted to consider prospective orientation, the participants were supportive.

“*… as a researcher we need to identify before we start the project, who takes the next step at the end of this, so it just doesn't end up being either a PhD thesis or a journal article…I don't see that that would be too onerous for us as researchers*” Researcher, MRI, 2016

The incentive that RIAFs could provide for researchers to engage with potential users and/or stakeholders at the research inception stage was strongly supported by participants. This view was presented from a policy, commercialisation, clinical practice, patient and ‘next’ researcher perspective.

A number of challenges were raised with prospective orientation. Firstly, while agreeing with its potential value, in the absence of embedding translation and impact into funding applications, track records, the grant review process and the wider culture of the general research institutions, the influence of an MRI RIAF was considered insufficient to drive this change alone.

“*People who are smart realising it* [a RIAF] *can be used more for assisting them in grant applications. But I think the driver always for grant applications will be whatever the criteria is for that grant, and not what we're expecting in terms of KPI for the Institute.*” COO, MRI, 2016

Secondly, researchers may be encouraged to engage with commercial partners to realise a RIAF metric, but in the experience of some participants, only the loss of commercial opportunities served to truly adjust their behaviour. A third challenge arose from the encouragement this objective provided for potential engagement with patients at formative stages of research. Participants reported good and bad experiences with patient representatives with respect to their contribution to the research process via symposiums, steering committees and meetings.

“*I have been in some meetings; air quality research which affects communities, steering committees; quite disruptive, haven't understood the scientific process, spend a whole lot of time explaining; they've got a particular barrow to push; we're trying to test the hypothesis and we'll come down on either side of it,* [while] *they've got a particular set view.*” Researcher, MRI, 2016

One participant noted that research already has to undergo ethics, budget and potentially sample size and health economics reviews prior to funding application. The introduction of a translation and impact review was considered complementary to this process.

#### Research quality control

Given that sustainable impacts can only be founded on high-quality research, participants were prompted to consider whether metrics reflecting research practice guidelines should be included in the impact framework. Examples from the literature include, where appropriate, systematic reviews, sample size calculations, study protocols, replication, publications of non-significant results, etc. Additional examples provided by the participants included mycoplasma-free laboratories, maintenance of digital IP-certified lab books, specific pathogen-free animal houses and data repositories.

“*I think absolutely it should be. The quality of research is essential in terms of ultimately achieving your goal of translation*” COO/Research Manager, MRI, 2016

“*I agree with that totally because if you can't rely on the science that's coming out then the whole thing's a waste.*” COO/Research Manager, MRI, 2016

A number of participants raised examples of problems, misconduct or quality control at their institutes demonstrating the prevalence of these challenges. While supporting the importance of quality control, other participants were less convinced that these issues should be addressed within a RIAF.

“*Researchers are very capable* [and] *would use their own skills/knowledge regarding best research practice and its relevance to research. Researchers have a wealth of knowledge. How much* [should be] *prescribed versus how much is personal? Don’t know.*” Researcher, MRI, 2015

Other participants described initiatives to address the problem with the intention of improving research quality, translation and ultimately impact.

“*No, I don't think you can really trust the researchers* [solely for research quality]*, but this is a battle that we wrestle with in a way. Part* [of the] *problem* [is] *people getting into it* [research] *for the first time. Our model, not formal, is to formulate big projects.* [As] *a novice researcher, you can come in and do this aspect of this big, well thought through, well-constructed project that's going on, rather than dreaming up your own little study; involved in a research project as a registrar or something and going to try and design a project. By the time they get it approved for ethics they've normally left.*” CEO, MRI, 2016

### Barriers to implementation of RIAFs

#### Engagement of basic science

The concern for basic science of a short-term political focus upon impacts was commonly raised as a potential barrier to engagement. Participants in management roles were typically more secure in the role of early stage science within a translation agenda, albeit the concern remained regarding uninformed government policy.

“*…if you’re impacting scientific thought and academic thought, I’m equally excited about that as I am if you’re impacting patient care and disease outcome.*” CEO, MRI, 2016

#### Conflicting incentives

Consistent with the existing challenges facing HMR, the main barriers presented regarding the implementation of RIAFs related not to their initial implementation, but rather their sustainability and relevance to researchers given conflicting incentives.

“*When you think about what the MRIs generally value, it is about papers and grants and things like that, and the grants in particular for very real reasons, because they're money in the door. You've got these strong metrics guiding people's behaviour, and really strong levers for reward. Then we're saying we want you to think much more about translation, but it's kind of like you've got to subscribe to the greater good, but it's not clear why you're doing that or what's the benefit for you, so I think there are real attitudinal barriers.*” KT Manager/Clinician Researcher, MRI, 2016

Beyond the publication/track record/grant cycle, researchers are exposed to additional incentives that potentially conflict and inhibit improved translation. Examples raised included academic career progression, which is founded largely on publication record, and teaching, which represents a dominant requirement for many academic roles.

“*…people in the university hate me for saying this, but the generation of new knowledge I think is secondary to the core purpose of universities which is teaching. The main output that universities have as a function of our community is qualified people.*” COO, MRI, 2016

#### Administrative burden

The fear of additional administrative burden was raised with respect to both the institutions and researchers. Administrative burden was presented as both the time to collect and report requisite translation and impact data/information, and concern that it will become another opaque requirement, such as ethics approval, which raises risks and consequently, resource requirements.

“*I've been doing this for 26 years, I can't believe how much time now we spend on ethics applications.*” Senior Researcher, MRI, 2016

It was noted that, for researchers to engage with and populate the data/information, the process has to carry value by building into researchers’ vision to make a difference.

“*The big test will be when we actually define the processes as to whether people see that this is of value or whether it's just another thing that I have to do to get my grant.*” Researcher, MRI, 2016

Finally, it was noted that a cost-effective data/information collection system was needed that did not duplicate data collected via other mechanisms, such as the Excellence in Research Australia, state government funding infrastructure reports, the National Survey of Research Commercialisation, etc.

“*…if they’re labour-intensive, that becomes a problem because that affects people’s productivity.*” Stakeholder, 2016

For other participants, it was important that the resource requirements were placed in context. Firstly, supporting resources should be considered in the wider context.

“*The onus is to cost a translation system, not just a framework.*” KT Manager/Clinician Researcher, MRI, 2015

Secondly, evidence was required to justify the resource commitment.

“*Need to have an economic evaluation of the cost of collecting data. NHMRC placed the onus on the researcher, not to be wasteful, to do this and that, there is a need to be accountable for the evidence base for implementation of these processes.*” CEO, MRI, 2016

#### Immaturity of field

While generally supportive, the respondents were cognisant of the paucity of evidence in this field.

“*We would think measurement is important and reporting is important,* [but] *there needs to be a much broader thinking on how we do this.*” Commercialisation Manager, MRI, 2016

“*What you're doing is actually translational research, which is ‘How do we best do translation?’, and I think that's very, very under done generally in the world and particularly in Australia.*” Clinician Researcher/KT Manager, MRI, 2015

### Methods – metrics

The attitude of participating MRIs towards the ‘measurement’ of research translation or research impact is multifaceted.

#### Measurement is important because…

Respondents believed that the measurement of research impact was “*tricky, but necessary*” (CEO, MRI, 2016). The capacity for data to inform decisions “*what gets measured, gets managed*” (COO, MRI, 2016) and for measurement to influence behaviour represented the most common reasons in support of measurement and metrics.

“*NHMRC a few years ago saying that it was going to count publications rather than the impact factor...the average impact factor of our publications went down as a result afterwards. Whatever you measure drives behaviours*.” COO, MRI, 2016

Participants also believed that the selected measures sent a clear message regarding the culture and direction of their respective institute.

A contrary view was presented by participants embedded within the case study tradition. From their perspective, engagement can be measured, but impact can only be assessed because no metrics can be consistently applied across all academic disciplines. A minority also believed that measurement of scientific output was a waste of time, that ‘clever’ scientists should be left alone, funded for 20 years and good things will happen. The view was presented by one participant, who also foresaw that this position was untenable in the current climate. Others simply favoured narratives because metrics were difficult.

The capability of metrics to influence behaviour was noted as a strength, but also a risk.

“*Measurement is really dangerous because all ways of measuring output and impact are imperfect, but they all drive behaviours.*” COO, MRI, 2016

“*I like the idea of frameworks to measure; I'm concerned about how that might then direct research too much; might guide government in directions that don't benefit society ultimately.*” Researcher, MRI, 2016

#### Issues with metrics

The majority of participants noted that the question of what to measure was critically important and remained a vexed issue. Measurements focussed upon the traditional academic outputs of publications, grants, etc. were seen as supporting existing behaviour and corresponding impacts. To improve health outcomes or generate wider societal benefits, the measurements needed to reflect outcomes, milestones or equivalent measures of translational progress, rather than outputs, such as publications or committee representation.

Participants noted that some measures were difficult to quantify, such as impact upon clinical practice or utilisation by policy-makers, international policy influence or any influence shaping views or wider societal impacts.

“*It really does come down to policy impact and service impact. We're not trying to measure the ultimate outcome, which is societal impact, because that's too long term; too difficult.*” Researcher, MRI, 2016

The concern existed that measurement may incentivise the easily measured rather than optimal translation.

The time lag between research and potential impact was regularly raised for both policy and basic science research, where the benefits may not be realised until the next generation; a period during which many other influences may cloud appropriate attribution. Related questions included how much ‘impact’ could be attributed to a given research output and the extent that any given research can claim to have ‘caused’ a given impact. How to measure what was not done because research excluded the option represented another participant concern.

Amid the context of potentially distant final health impacts, the potential for leading indicators of impact was raised by several participants.

“*You've got to create a framework which gives you those lead indicators as to whether somebody is on to something.*” COO, MRI, 2016

However, some respondents were concerned that the use of interim process metrics could undermine the objective.

“*I’m wary of perverse incentives with process metrics, e.g. if researcher targets clinical guidelines, and focus upon attendance on guideline committees, but guidelines are not being implemented? How do you get the behaviour, need to be really careful to encourage.*” CEO, MRI, 2016

Engagement metrics, such as those proposed within the Research Engagement Australia model [16], were considered as process metrics by some. One participant anticipated that engagement with industry and other users might rise as a result of these metrics, but was not convinced that this would translate to greater impact. Measurement of both interim and final metrics was seen as a potentially valuable tool to understand the leading indicators of impact.

Others emphasised that standardised metrics remain important.

“*Process metrics fine, but you've still need something which enables you to compare apples with oranges across the institute. Management needs to decide whether to fund* [an] *epidemiological project in the NT with Aboriginal people or a piece of cell biology in the lab on the fourth floor.*” COO, MRI, 2016

Discussions also introduced the challenge that appropriate measures may vary depending upon the research and the potential impact. For example, respondents to the National Survey of Research Commercialisation found it difficult to accommodate complicated commercial arrangements within a standard framework. A view existed that metrics should be appropriate to the research and the research translation/impact pathway, rather than standardised for the sake of standardisation. However, customised metrics should be meaningful and validated.

“*I guess tailored metrics are okay. Ultimately want to measure ‘benefit’, rather than gamesmanship…They* [process metrics] *need to be reviewed according to capacity to be ‘gamed’.*” CEO, MRI, 2016

A requirement was noted for different metrics that could reflect the facilitation of research translation. For example, the number of researchers operating in applied roles, be this clinical practice, public health or similar, was considered to increase the likelihood of translation.

“*Half our staff work there* [Cardiology Department, Hospital]*, or half of their staff are also employed by us, then you've got better people doing better work which is more informed by best practice around the world as well as by their own research; readmission rates are lower, clinical outcomes are better, survival rates post separation are better.*” COO, MRI, 2016

Other potential facilitation metrics related to the evidence required to assess whether initiatives to improve translation are proving effective, including engagement with biostatisticians, industry/government engagement strategies, etc.

#### How to analyse

The question of how to analyse metric data was rarely raised, but for a few participants it represented as a critical issue with respect to the relevance and potential insights provided. For example, the aggregation of Research Engagement Australia’s metrics was also considered to provide minimal differences and insight, save for the larger universities. The choice of metric and the form of analysis can affect the insights and/or the capacity for the institution to demonstrate improvements.

#### Unit of analysis

Many of the participants intertwined discussions of appropriate measures with either individual or institutional KPIs, but failed to question the appropriate scale of analysis. The optimal unit of analysis was raised by one participant, who argued that assessment should be conducted at the research project level, rather than for individuals, to ensure that the incentives are aligned with research collaboration and shared initiative that reflect research practice and considered optimal for translation.

### Methods – case studies

The participants held clear views regarding the respective strengths and weaknesses of case study methods for the assessment of research translation and research impact.

#### Strengths

The value of case studies for advocacy purposes was presented by numerous participants. Clearly articulated case studies were appreciated as effective communication tools for corporate donors, philanthropists and politicians. They were recognised as a more complete picture of research, particularly when complementing research metrics. Case studies could similarly explain a more nuanced research story and present significant outcomes that could not be readily quantified such as paradigm shifts in knowledge. Another advantage presented for this method included the belief that smaller research institutions were not disadvantaged in their capacity to realise and demonstrate impact. One respondent also valued case studies as a change management agent for engaging researchers in thinking about translation and impact more explicitly.

#### Weaknesses

The discussions also highlighted perceptions of many weaknesses and limitations of this method. Criticisms by senior politicians of case studies, as ‘telling stories and anecdotes’ were raised, inferring that the benefits for advocacy may not be comprehensive. While peer-review and strong guidance can manage research claims and limit exaggeration, the process to audit and validate case studies was perceived as challenging. Participants also raised concerns with the administrative burden for researchers and research administration. One stakeholder noted that the RAND Europe review of the United Kingdom’s Research Excellence Framework, a peer-review case study method, was estimated to cost 2% of total research expenditure, which they perceived as reasonable. Others were concerned that the burden would fall on researchers and questioned whether the benefits justified the additional distraction.

It was argued that the knowledge of the intended readership also acted as a constraint on the capacity of case studies to represent a more sophisticated impact story. Other participants were also less convinced that smaller institutions would not remain disadvantaged between spikes of exemplary impact.

The strongest criticism of the case study method arose with selection bias. The presentation of positive examples and examples that can be readily explained represented a significant limitation.

“*You don't case study all the stuff that's not working and really when you're doing…metric analysis you need to be looking at what's not working…to rectify things and change your orientation.*” CEO, MRI, 2016

### Methods – economic assessment

Respondents were asked whether they see a role for economic evaluation and the nature of any prior economic evaluations. A few participants acknowledged a lack of understanding of economic methods and their potential relevance to research impact assessment. Of those with a view, economic assessment was variously interpreted as health economic studies or techniques, health needs/services assessment, commercial market evaluation, or macroeconomic assessment of HMR.

#### Health economic studies

Health economic studies were seen as beneficial in the capacity for the evidence, often founded upon experimental research, to translate new health interventions beyond economic hurdles, e.g. Pharmaceutical Benefits Advisory Committee reviews, or resource constraints, e.g. budget sustainability of policy changes or service programmes. Some institutes reported that process and economic evaluations were required for all health service projects. Two institutes advised that they were considering pre-study cost-effectiveness analyses for relevant interventions. The intention of prospective cost-effectiveness analyses is to inform upon the probability of passing an economic hurdle. If the probability is low, the likelihood of translation and impact will be correspondingly low and, without alternative justification, the study may change or the expenditure would prove more effective if invested in research with a higher prospect for impact. Health economics techniques, such as the derivation of Quality-Adjusted Life Years were pictured as a method to provide a direct estimate of the health impact of research.

#### Health needs/service assessment

In a broader sense, health economic assessment was interpreted as a method to identify areas of health needs/health service assessment, where targeted research could potentially translate to significant impacts.

#### Commercial market evaluation

Three participants with a commercialisation focus considered economic assessment to be commercial market evaluation. These evaluations examine the potential market size, competitors, regulatory hurdles, intellectual property issues and revenue sources, such as Medicare or private health insurance, in advance of decisions to invest in additional research.

#### Macroeconomic assessment of HMR

The view was presented that sector-wide economic analyses of the benefits from medical and health research were unreliable, but provided useful material for advocacy purposes.

#### Challenges arising with economic assessment

A number of concerns were raised regarding economic assessments. Validity concerns arose from the potential for researchers to exaggerate anticipated benefits and, consequently, the economic returns from their research. The financial viability of conducting economic evaluations on all projects was also questioned. For some, this implied that economic assessments were more appropriate for research with readily monetised benefits. The reliance that an economic analysis may place upon a direct link between anticipated benefits and investment in the research was considered unrealistic for some research with the concern that this may reduce investment in potentially impactful research.

“*Take Gardasil, the returns would probably pay for 20 years of NHMRC research expenditure. In that sense we’ve used* [this example] *to say to government,* [HMR is a] *‘good place to invest’. The big problem is the direct relationship that everyone seeks.*” Stakeholder, 2016

Similar concerns were raised that economic assessment would discourage research of rare diseases with small populations.

### Utilisation and end-users

The failure to engage end-users at the conception stage of research was raised as a failing for both commercialisation, health system and policy research.

“*It is very hard to market IP* [intellectual property]*, where an external party/company has not been involved in its development.*” COO/Researcher, MRI, 2015

Co-production or early engagement with ‘users’ of the research was presented as one method to address this challenge.

“*Very early stakeholder engagement even defines what the problem is and then what potential solutions might exist is crucial. We want to make that more systematic, that engagement at the beginning, end user processes to design interventions, engaging stakeholders throughout the research process, and then, of course, engaging in that advocacy process at the end of the research often in partnership with those relevant stakeholders.*” Researcher, MRI, 2016

“*That doesn't sound too onerous and it would seem like it would give some clarity to researchers, because ultimately we do want to do stuff that can be replicated, people can have confidence in, that builds our standing in the community and that does actually make a difference.*” Researcher, MRI, 2016

The engagement and development of clinician researchers was seen as another method to address this challenge.

“*Spending two sessions a week with patients/running practice,* [your] *chances are better at spotting* [where the] *translational impact of work is going to come from.*” COO, MRI, 2016

If not clinicians, policy-makers or industry, the end-users for basic science were nominated potentially as other basic scientists. However, it was considered important that, even if the next ‘user’ might be a researcher, the final impact should remain central to basic scientists’ research goals.

“*I would hate the basic scientists to think that they can simply gain approval for their work from fellow experts. Consideration of the final objective can heavily inform the focus of their work. For example, studies on patients suffering from rheumatoid arthritis found that fatigue, rather than pain…dominated their priorities. This consideration was novel to many basic scientists and should inform on the focus of their research.*” CEO, MRI, 2016

Examples were provided of funding mechanisms that could drive engagement with commercial users.

"*Possibility to resolve with principal funding, e.g. health market validation fund (Victorian Government halted, potentially reinitiated soon); where companies bid funding to resolve problems, the companies subsequently sub-contract to the researchers.* [The] *NSW Government equivalent medical device fund works well."* COO/Commercialisation Manager, MRI, 2015

### Commercialisation

The issues regarding the assessment of research commercialisation demonstrate how measurement is closely integrated with both existing and anticipated practice. The following sub-themes were identified from the interviews.

#### Attitude to commercialisation

Numerous participants expressed the view that commercialisation was often essential to translate research into health impacts. None of the participants presented an overtly negative perspective of commercialisation, although numerous challenges and conflicts were identified. Only two participants alluded to economic impacts as an explicit outcome, although several valued the revenue generated from these ventures, given the capacity this provided for untied direct investment into their research programmes. Alignment of commercialisation with the health goals of the institute was considered imperative by two participants.

“*If you don't make much money out of it that's a shame, but it's much better than not getting it out there and not having it used.*” COO, MRI, 2016

Finally, it was noted that, outside Australia, the populations of emerging nations often rely upon private healthcare systems. Consequently, commercialisation was seen as an important pathway to ‘scale up’ and optimise health impact in these health systems.

#### Existing policies

The MRIs reported a significant range of sophistication with regard to commercialisation policies and processes. Some of the institutes were highly advanced in their initiatives to encourage commercialisation. Their capabilities included explicit commercialisation committees to guide governance; dedicated roles for business development; establishment of discrete, fully-owned entities with separate management and objectives to conduct and manage commercial research, particularly clinical trials for commercial clients; comprehensive incentive programmes for researchers for all progressions along the intellectual property (IP) protection path; generous IP policies, positioned advantageously to equivalent university policies, and usually founded upon technology transfer to the investigator, while recovering the costs of establishing any IP vehicle; indenture-ship rewards from successful deals; and funding initiatives to enable ‘blue sky research’, address early ‘valleys of death’, such as proof of concept studies, or to enable the conduct of data collection for commercial purposes, such as developing assays to generate investor-ready data. The commercialisation approach in one institute also extended to good commercial practice, in areas such as laboratory notebook standards, to ensure counter-signatures and compliance with international IP law.

At a minimum, MRIs defaulted to commercialisation policies held by associated universities. The incentive systems provided by some institutes also appeared to inhibit commercialisation.

*“The policy is whatever you do when you're here on our time is ours…It isn't very conducive to encouraging that”* [commercialisation]. Researcher (directly employed), MRI, 2016

The majority of participants presented the approach to commercialisation by their institution somewhere between these extremes. Commercialisation processes exist, access to commercialisation experts was usually possible and industry engagement was encouraged, but for most institutes a formal and comprehensive approach to commercialisation did not exist.

#### Problems/challenges in commercialisation

A number of sub-themes were raised regarding challenges associated with commercialisation such as the difficulty to source research or industry funds for early phase clinical trials. As noted prior, some of the institutes invested their own funds to overcome this hurdle. The conflict between academic and commercial objectives was also commonly raised. The protection of commercial IP potentially conflicts with the timing of peer-review publication and delays in publication potentially cascade into a weaker academic track record for grant applications. The incentive for researchers consequently remains to prioritise academic outputs above, rather than parallel to, commercial translation. Prioritisation of commercialisation by researchers was noted to generate significant risks.

“*I have seen and can count, more than a handful of really high level, driven intelligent quite senior researchers fall into the trap where they have become very translational and quite successful in terms of … technologies or … industry trials …but have less and less – away from that core research. Once that side of things reaches maturity … where the research contribution is now no longer needed because it’s moved on to another level of development, they fall into the trap where they’re basically in no man's land. Unfortunately, I have seen a lot of them have had to leave the industry. I find it very, very sad because I have personally had to deal with this a number of times.*” Commercial Development Executive, Medical Research Institute, 2016

Many participants noted institutional support for engagement with industry and commercialisation activity, but many also questioned whether the incentives were sufficient to draw intellectual focus away from pure publication. For research with commercialisation opportunities the restrictions regarding the capacity to pay for patent attorneys, etc. was identified as an inhibitor.

While commercialisation metrics were raised by participants, the focus of the discussions remained with the extent to which commercialisation is effectively integrated into the research process. As such, it demonstrates how RIAFs were seen as both a means to bring transparency to the strengths and weaknesses of current practice, and a means to actually improve commercialisation and the according potential for improved research impact.

## Discussion

Policy regarding impact assessment of the funds invested into Australian health and medical research continues to progress rapidly. For example, in response to demands for impact assessment from the Australian Federal Government, the Australian Research Council piloted an Engagement and Impact Assessment of Australian universities in 2017 [17], intended to complement the existing assessment framework for research quality and excellence (Excellence in Research Australia [18]). The parallel health-focussed organisation, the Australian National Health and Medical Research Council, has not formalised a research impact assessment framework, but has undertaken numerous initiatives to progress research translation, including the establishment of the Advanced Health Research and Translation Centres and Centres for Innovation in Regional Health [19]. The NHMRC’s policy position regarding research impact assessment is presently under development. Philanthropic funders have also accelerated their work with grant recipients to improve the evaluation of research and ultimately the impact generated from their significant investment into MRIs across Australia [20].

The imperatives driving the push from research funders to implement research impact assessment are unlikely to wane. Consequently, there is an increasing probability that such assessment will be implemented for the HMR conducted within Australia’s MRIs, either via the demands of external funders or through the MRIs’ own initiatives. As such, it is necessary that the design of any frameworks and assessment systems are well-grounded in the practices, concerns and motivations of health and medical researchers and the institutional framework that supports their work. This research sought to increase transparency regarding the views of stakeholders, researchers, research managers and strategic leaders of Australia’s MRIs regarding research impact assessment.

The first aim for this qualitative research sought to elicit the views of MRIs regarding objectives, definitions, methods, barriers, potential scope and attitudes towards RIAFs. A number of overarching issues can be highlighted from the subject themes identified in the framework analysis.

The first overarching issue relates to the strengths and weaknesses of the existing research environment with respect to the generation of impact. Those interviewed noted numerous examples regarding how existing incentives within funding and promotion frameworks conflicted with the generation of greater impact. For example, the commercialisation of IP comprises a valuable step towards the realisation of economic and/or health impacts. However, some participants believed that the peers reviewing funding applications for research with a commercial component rarely possess the knowledge or experience to assess the focus, approach or potential contribution of research in this light. The Australian NHMRC has recently taken steps to address this concern through advertised recruitment of commercialisation expertise to the assessment committee for the Development Grants scheme. Similar limitations were reported for implementation within the health service. Initiatives, such as the establishment of the NHMRC Advanced Health Research and Translation Centres/Centres for Innovation in Regional Health, continue to be developed and implemented to address this weakness in the translation process.

A second overarching issue relates to the fundamental purpose of research impact assessment, given the objective holds implications for the form and method of assessment. If accountability or assistance of management decisions through benchmarking and transparency represents the intention, then the methods can be tailored to realise this goal. However, if the objective is to incentivise and change behaviour to realise improved health outcomes and other social/economic benefits, then this potentially holds a range of implications for impact assessment. For example, the unit of analysis needs to be sufficiently granular for research teams to gain attributable credit for realising progress towards potentially impactful goals. This question has not been adequately addressed in Australia’s approach, nor the approach of many international frameworks.

A third overarching issue relates to the numerous technical questions that require clarification. These considerations include agreements regarding definitions, manageable approaches to attribution, causation and time lags, appropriate methods, choice of indicators, optimal economic methods for different research types, as well as systems issues relating to administrative efficiency, quality control and researcher engagement. The results confirm that many of the impact assessment issues identified by RAND [21], the Australian Research Council [22] and the Canadian Academy of Science [23] remain prevalent concerns within the MRI community. However, the results substantially broaden these considerations to place research impact assessment and measurement within the context of how the system shapes existing research activity and, consequently, how research impact assessment may augment or undermine the generation of greater impact.

The second research aim sought to question the perceived value of a prospective approach to research impact assessment, in contrast to the commonly practiced retrospective approaches. The research found that participants implicitly assumed impact assessment to be a retrospective process. The utilisation of impact assessment frameworks to guide, inform and prospectively optimise research *ex ante* from inception to an ex post reflection represented a novel consideration. The majority of participants were supportive of the rationale, but raised questions regarding the mechanisms through which prospective assessment would be implemented. The opportunity that this approach presents for impact assessment merits further investigation, including the initiatives pursued by Searles et al. [13], Graham et al. [24], Tsey et al. [25], Trochim et al. [26], Herbert et al. [27] and Greenhalgh et al. [28], as it potentially holds implications for the methods and the systems required to support this approach.

The results of this analysis carry numerous potential implications for the prospective research impact assessment of HMR both within Australia and internationally. These implications are expanded within an accompanying paper regarding the guiding principles for prospective RIAFs within MRIs.

The overarching objective for this research was to inform upon the development of guiding principles for a RIAF by eliciting the views of Australia’s MRIs regarding research impact assessment and alternative approaches to this assessment. We found a willingness amongst the respondents to implement RIAFs and acknowledgement of the factors pressing for their implementation. However, the respondents also raised numerous concerns with research impact assessment. These issues need to be addressed by the guiding principles if impact assessment is to contribute to the over-arching goal, namely the optimisation of impacts from investment in HMR.

This study contains a number of limitations. The purposive sample is not assumed to reflect a representative sample of the Australian MRIs nor Australia’s wider HMR community. However, for this stage of policy development, diversity of opinion was prioritised over generalisation. The sample number was not determined by theme saturation. Some selection bias might have been introduced to the study given those interviewed potentially represented researchers and research management with greater knowledge and insight into research policy. The views of those less informed and potentially less supportive of research impact assessment may be under-represented. The intention of the research was to identify relevant issues, it cannot attribute relative significance beyond the perceptions of the respondents.

## Conclusion

The stated purpose for most of Australia’s MRIs is for the research conducted within their remit to improve the health of individual patients and the collective population. However, consensus is lacking regarding the best methods to assess progress towards this goal. This qualitative study found that the issues raised by Australia’s MRIs regarding research impact assessment are less about methods and metrics, and more about the research activities that the measurement of research translation and impact may or may not incentivise. Consequently, if impact assessment is to contribute to optimisation of the returns from the public, corporate and philanthropic investment entrusted to the institutes, then an understanding of how the process may re-align research behaviour must be prioritised. The insights drawn from this research must be addressed in the development of a RIAF for Australia’s MRI community.

## Appendix 1

The following medical research institutes participated in this research:

- Baker IDI Heart and Diabetes
- Bionics Institute of Australia
- Burnet Institute
- George Institute for Global Health
- Kirby Institute
- Mater Research/Translational Research Institute
- Menzies Research Institute Tasmania
- Murdoch Children’s Research Institute
- National Ageing Research Institute
- QIMR Berghofer
- Sax Institute
- SAHMRI
- Telethon Kids Institute Perth
- Walter & Eliza Hall Institute of Medical Research
- Woolcock Institute of Medical Research

Participating stakeholders:

- Australian Association of Medical Research Institutes (policy insight)
- National Health and Medical Research Council (funding body)
- Australian Research Council (funding body)
- Dr. Brent Jenkins (commercialisation)
- University Research Director/Consultant – In confidence (International and Australian research impact assessment context)

The views provided by employees of these organisations may not reflect the views of the employer.

## Appendix 2

A semi-structured interview guide was used to structure the discussion. The semi-structured approach provided consistent coverage of key issues, while allowing for the interviewers to encourage respondents in new areas of investigation.

**SEMI-STRUCTURED INTERVIEW GUIDE – HMRI-DOIIS Research Impact Project.**

**Interview with [INSERT INDIVIDUAL/S], [INSERT INSTITUTE].**

Contact No. XX XXXX XXXX FOR TELECONFERENCE.

SURVEY START.

Introduction

- Purpose of the research project, for whom, what will be done with the research

Approval to record

- Do you mind if we record this interview?

1. While we have investigated the Institute’s public information, could you please provide a brief summary of the organisation’s structure?
  - Relationship to researchers – Employer, facilitator?
  - Research spectrum – Basic science, clinical, population health?
  - Scale
  - Funding attributes that differ to the usual MRI profile (example?)
2. What is your role within the organisation?
3. What is your understanding of the term ‘research translation’? Does your institute have a clear definition?
4. What is your understanding of the term ‘research impact’? Does your institute have a clear definition?
5. What is your understanding of the term ‘knowledge transfer’?
  - If so, how does it differ from the above
6. What is your attitude/belief toward the measurement of research translation/research impact?
7. Does the organisation presently evaluate research other than via the Excellence in Research Australia metrics?
  - If so, how?
8. Does your organisation presently have a framework and/or metrics designed to measure research translation/research impact? (Note: These questions will apply to a small proportion of the sample population)
  - If yes:
    i. What is the purpose or objective of your existing evaluation framework? (accountability, advocacy, learning and feedback)
      1. If prospective change of the research process is not noted, ask for their opinion
    ii. Could you please describe the framework/metrics?
    iii. From your perspective, how would you describe the implementation/operation of the framework/metrics?
    iv. From your perspective, how has the framework/metrics succeeded and/or failed in realising the intended objectives
  - If no:
    i. Do you see a need or a role for Research Impact Frameworks?
      1. If Yes go to Q9
      2. If No – On what grounds do you hold that opinion?
    ii. Accountability, advocacy, learning and feedback are regularly provided as reasons for the assessment of research impact. The potential exists to use the framework to encourage good research practice in the same manner that Excellence in Research Australia seeks to encourage good quality? Do you have a view as the capacity for research impact metrics to effect such change?
9. What are the problems with current medical and health research practice that should be addressed? Why change anything?
  - Do you see a role for Research Impact Frameworks in addressing these challenges?
10. From your perspective, what are the potential barriers to measuring research translation/research impact?
  - Issues relating to burden/administration
  - Issues pertaining to instilled changes to perverse incentives and alike
11. Case studies:
  - If an evaluation framework exists for the organisation’s research:
    i. Do case studies play a role?
    ii. Strengths/weaknesses of case studies
12. If an evaluation framework exists for the organisation’s research:
  - Does it contain metrics/indicators?
  - Are the metrics published?
  - How were the metrics determined?
  - Have the metrics been evaluated to determine whether they realise their objective?
13. Checklists and guides exist to optimise the value from research investment. An example is the template for Intervention Description and Replication (TIDieR) checklist and guide:
  - Does your Medical Research Institute (MRI) subscribe to any such research practice guidelines as policy? Or is it left to the researcher?
  - Do you think that the MRI specifically has a role in encouraging best practice that optimises the value of research investment?
14. Do you have policies to encourage commercialisation? Do these policies have associated metrics?
15. Turning to the systems and work practices established to collect indicators?
  - Are metrics currently collected? Consistently or periodically?
  - Are systems in place to efficiently collect metrics?
  - Are metrics collected prospectively?
16. Economic evaluation
  - Do you see a role for economic evaluation of research?
  - Have economic evaluation’s been conducted on the MRI’s research?
    i. How are these reported?
    ii. What is the research scope of these evaluations? Project, grant, researcher, programme, administrative unit?
17. [IF TIME]: The Hunter Medical Research Institute have developed an initial impact assessment framework called FAIT, comprising a combination of case studies, metrics and economic evaluation (elaborate as required)? The measures include process metrics and encourage prospective implementation. What is your attitude towards this approach to measuring research translation/research impact?
18. Do you have any other comments?

Thank you.

If you have any further questions, or you would like to reflect and adjust your opinion on any of the above, or to add any further comments, please feel free to contact me via the contact details provided.

SURVEY END.

## Abbreviations

CEO: Chief Executive Officer
COO: Chief Operating Officer
FAIT: Framework To Assess the Impact from Translational health research
HMR: Health and Medical Research
IP: intellectual property
KPI: Key Performance Indicator
KT: knowledge transfer/translation
MRI: Medical Research Institute
NHMRC: National Health and Medical Research Council
RIAF: Research Impact Assessment Framework
